# 2′-Amino-3,6-dihydroxyxanthene-9-spiro-1′-isoindolin-3′-one monohydrate

**DOI:** 10.1107/S1600536808000032

**Published:** 2008-01-09

**Authors:** Dong-Xiang Wang, Gen-Hua Wu

**Affiliations:** a School of Chemistry and Materials Science, Anhui Normal University, Wuhu 241000, People’s Republic of China; b School of Chemistry and Chemical Engineering, Anqing Normal College, Anqing 246003, People’s Republic of China

## Abstract

The title compound, C_20_H_14_N_2_O_4_·H_2_O, was synthesized by the reaction of fluorescein and hydrazine hydrate in ethanol. In the crystal structure, the organic mol­ecules are linked into extended two-dimensional networks by inter­molecular hydrogen bonding. Additional face-to-face π–π stacking inter­actions between the phenolic benzene rings in two adjacent mol­ecules [centroid-to-centroid separation = 3.773 (3) Å] link the mol­ecules into a three-dimensional framework.

## Related literature

For general background, see: Chen *et al.* (2006 ▶); Yang *et al.* (2005 ▶); Adamczyk *et al.* (2000 ▶). For related literature, see: Orndorff *et al.* (1927 ▶).
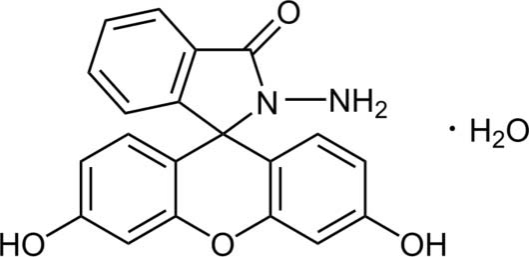


## Experimental

### 

#### Crystal data

C_20_H_14_N_2_O_4_·H_2_O
*M* *_r_* = 364.35
Triclinic, 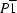

*a* = 7.8524 (9) Å
*b* = 10.7077 (13) Å
*c* = 11.2137 (13) Å
α = 103.857 (2)°
β = 110.432 (2)°
γ = 99.704 (2)°
*V* = 824.22 (17) Å^3^
*Z* = 2
Mo *K*α radiation
μ = 0.11 mm^−1^
*T* = 293 (2) K
0.32 × 0.26 × 0.22 mm

#### Data collection

Bruker SMART APEX CCD area-detector diffractometer
Absorption correction: multi-scan (*SADABS*; Bruker, 1997 ▶) *T*_min_ = 0.967, *T*_max_ = 0.977
4192 measured reflections
2892 independent reflections
1975 reflections with *I* > 2σ(*I*)
*R*_int_ = 0.020

#### Refinement

*R*[*F* ^2^ > 2σ(*F* ^2^)] = 0.050
*wR*(*F* ^2^) = 0.161
*S* = 1.01
2892 reflections
256 parameters
2 restraints
H atoms treated by a mixture of independent and constrained refinement
Δρ_max_ = 0.23 e Å^−3^
Δρ_min_ = −0.21 e Å^−3^

### 

Data collection: *SMART* (Bruker, 1997 ▶); cell refinement: *SAINT-Plus* (Bruker, 1997 ▶); data reduction: *SAINT-Plus*; program(s) used to solve structure: *SHELXS97* (Sheldrick, 2008 ▶); program(s) used to refine structure: *SHELXL97* (Sheldrick, 2008 ▶); molecular graphics: *XP* (Bruker, 1997 ▶); software used to prepare material for publication: *SHELXTL* (Bruker, 1997 ▶).

## Supplementary Material

Crystal structure: contains datablocks I, global. DOI: 10.1107/S1600536808000032/at2528sup1.cif

Structure factors: contains datablocks I. DOI: 10.1107/S1600536808000032/at2528Isup2.hkl

Additional supplementary materials:  crystallographic information; 3D view; checkCIF report

## Figures and Tables

**Table 1:** Hydrogen-bond geometry (Å, °)

*D*—H⋯*A*	*D*—H	H⋯*A*	*D*⋯*A*	*D*—H⋯*A*
O4—H4*A*⋯O1*W*^i^	0.82	1.99	2.790 (3)	165
N1—H1*B*⋯O1^ii^	0.89 (3)	2.534 (16)	3.025 (3)	115.4 (19)
O2—H2⋯O1*W*^iii^	0.82	1.95	2.760 (3)	170
O1*W*—H1*WA*⋯N1	0.85	2.23	2.883 (3)	134
O1*W*—H1*WB*⋯O1^ii^	0.87	2.06	2.861 (3)	152

Symmetry codes: (i) 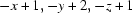; (ii) 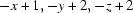; (iii) 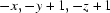.

**Fig. 1.**
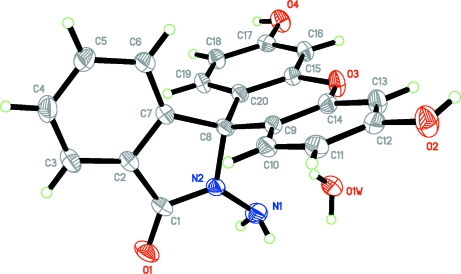
The asymmetric unit, with the atom-numbering scheme. Displacement ellipsoids are drawn at the 30% probability level.

**Fig. 2.**
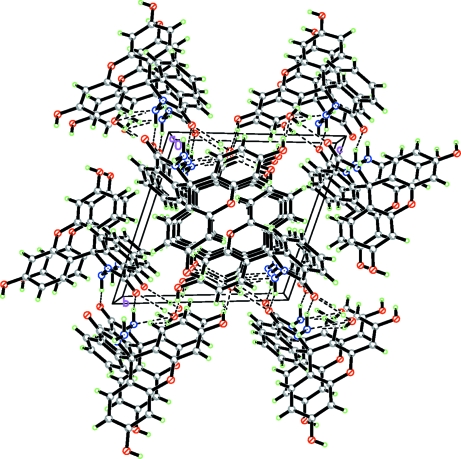
A packing diagram for (I). Hydrogen bonds are shown as dashed lines.

## supplementary crystallographic information

### Comment

The development of fluorescent probes for determining various analytes with high selectivity and sensitivity has attracted much attention in recent years (Chen, *et al.*, 2006). So, an enormous amount of research has gone into the design and synthesis of fluorescent probes. Fluoresein is one of the most popular dyes, because fluorescein has many advantages, including high fluorescence quantum efficiency, high extinction coefficient around 490 nm, and high water solubility under physiological conditions, therefore, it is usually utilized as reporting group in routine optical analysis (Yang *et al.*, 2005; Adamczyk *et al.*, 2000). For example, The title compound can be a probe to detect copper(II), cobalt(II) and hydrogen peroxide. This promoted us to attempt to prepare and obtain the crystals of the other fluorescein derivatives and characterized their crystal structures. In the title compound, C_20_H_14_N_2_O_4_.H_2_O, termed "fluorescein hydrazide", was prepared by reaction of fluorescein with hydrazine hydrate. Although fluorescein hydrazide has been reported by others, there is no report about the crystal of fluorescein hydrazide suitable for single-crystal X-ray diffraction. Herein, we report the crystal structural details on fluorescein hydrazide.

The fluorescein hydrazide was confirmed to have a five-membered spirolactam structure. The spiro form fluorescein hydrazide bearing a cleavable active bond is characterized by single-crystal X-ray diffraction.

The asymmetric unit contains one organic molecule and one water molecule. The benzene ring of phenol deviates only slightly from planarity with a dihedral angle of 10.18 (3)°. The water O atom acts as a hydrogen bond acceptor and donor from the hydroxy group in a neighouring organic molecule, thereby forming extended 2-D networks (Table1, Fig.2). The crystal packing is characterized by π···π stacking interactions. The molecules are stacked in an antiparalled fashion, with phenyl ring of phenol centroid-centroid separation of 3.773 (3) Å. Together with the hydrogen bonds, these interactions lead to a three-dimensional supramolecular network pattern (Fig. 2).

### Experimental

For the synthesis of fluorescein hydrazide, different procedures have been reported (Orndorff *et al.*, 1927). In this work, a modified literature procedure was used to produce fluorescein hydrazide. A solution of fluorescein (1.0 g, 3.0 mmol) in absolute ethanol (50 ml) was stirred and 4.0 ml (excess) hydrazine hydrate (85%) was then added dropwise with vigorous stirring over 5 minutes. The solution was refluxed for 5 h. The reaction mixture was cooled and the solvent was removed under reduced pressure to give dark orange oil. Then, 30 ml of ethanol/water (v:v = 7:3) was added to the oil, a light orange crystal suitable for single-crystal *X*– ray diffraction was obtained by evaporating the resulting solution in air for several days. The resulting light orange crystal was filtered, washed with ethanol, and then dried in vacuo, affording of the title compound [yield: 0.98 g, 90%]. The product is stable in air.

### Refinement

All H atoms were positioned geometrically (C—H = 0.93 Å and O—H = 0.82 Å), and refined using a riding model, with *U*_iso_(H) = 1.2*U*_eq_(C or O).

### Crystal data

**Table:**

C_20_H_14_N_2_O_4_·H_2_O	*Z* = 2
*M**_r_* = 364.35	*F*_000_ = 380
Triclinic, *P*1	*D*_x_ = 1.468 Mg m^−^^3^
Hall symbol: -P 1	Mo *K*α radiation λ = 0.71073 Å
*a* = 7.8524 (9) Å	Cell parameters from 988 reflections
*b* = 10.7077 (13) Å	θ = 2.4–24.3º
*c* = 11.2137 (13) Å	µ = 0.11 mm^−^^1^
α = 103.857 (2)º	*T* = 293 (2) K
β = 110.432 (2)º	Block, light orange
γ = 99.704 (2)º	0.32 × 0.26 × 0.22 mm
*V* = 824.22 (17) Å^3^	

### Data collection

**Table:**

Bruker SAMRT Apex CCD area-detector diffractometer	2892 independent reflections
Radiation source: sealed tube	1975 reflections with *I* > 2σ(*I*)
Monochromator: graphite	*R*_int_ = 0.020
*T* = 293(2) K	θ_max_ = 25.1º
phi and ω scans	θ_min_ = 2.0º
Absorption correction: multi-scan(SADABS; Bruker, 1997)	*h* = −9→8
*T*_min_ = 0.967, *T*_max_ = 0.977	*k* = −12→12
4192 measured reflections	*l* = −11→13

### Refinement

**Table:**

Refinement on *F*^2^	Secondary atom site location: difference Fourier map
Least-squares matrix: full	Hydrogen site location: inferred from neighbouring sites
*R*[*F*^2^ > 2σ(*F*^2^)] = 0.050	H atoms treated by a mixture of independent and constrained refinement
*wR*(*F*^2^) = 0.161	*w* = 1/[σ^2^(*F*_o_^2^) + (0.0787*P*)^2^ + 0.372*P*] where *P* = (*F*_o_^2^ + 2*F*_c_^2^)/3
*S* = 1.01	(Δ/σ)_max_ < 0.001
2892 reflections	Δρ_max_ = 0.23 e Å^−^^3^
256 parameters	Δρ_min_ = −0.21 e Å^−^^3^
2 restraints	Extinction correction: none
Primary atom site location: structure-invariant direct methods	

### Special details

**Table:**

Geometry. All e.s.d.'s (except the e.s.d. in the dihedral angle between two l.s. planes) are estimated using the full covariance matrix. The cell e.s.d.'s are taken into account individually in the estimation of e.s.d.'s in distances, angles and torsion angles; correlations between e.s.d.'s in cell parameters are only used when they are defined by crystal symmetry. An approximate (isotropic) treatment of cell e.s.d.'s is used for estimating e.s.d.'s involving l.s. planes.
Refinement. Refinement of *F*^2^ against ALL reflections. The weighted *R*-factor *wR* and goodness of fit *S* are based on *F*^2^, conventional *R*-factors *R* are based on *F*, with *F* set to zero for negative *F*^2^. The threshold expression of *F*^2^ > σ(*F*^2^) is used only for calculating *R*-factors(gt) *etc*. and is not relevant to the choice of reflections for refinement. *R*-factors based on *F*^2^ are statistically about twice as large as those based on *F*, and *R*- factors based on ALL data will be even larger.

### Fractional atomic coordinates and isotropic or equivalent isotropic displacement parameters (Å^2^)

**Table:**

	*x*	*y*	*z*	*U*_iso_*/*U*_eq_	
C1	0.7582 (4)	0.8546 (3)	1.0078 (3)	0.0383 (7)	
C2	0.8972 (4)	0.7821 (3)	0.9920 (3)	0.0363 (7)	
C3	1.0760 (4)	0.7941 (3)	1.0832 (3)	0.0480 (8)	
H3	1.1261	0.8547	1.1699	0.058*	
C4	1.1785 (4)	0.7131 (3)	1.0418 (3)	0.0537 (9)	
H4	1.2993	0.7193	1.1012	0.064*	
C5	1.1019 (4)	0.6226 (3)	0.9121 (3)	0.0514 (8)	
H5	1.1725	0.5688	0.8859	0.062*	
C6	0.9215 (4)	0.6111 (3)	0.8207 (3)	0.0425 (7)	
H6	0.8709	0.5510	0.7337	0.051*	
C7	0.8206 (4)	0.6910 (3)	0.8629 (3)	0.0342 (6)	
C8	0.6212 (4)	0.6958 (2)	0.7840 (2)	0.0323 (6)	
C9	0.4728 (4)	0.5651 (3)	0.7417 (2)	0.0322 (6)	
C10	0.4807 (4)	0.4862 (3)	0.8257 (3)	0.0375 (7)	
H10	0.5825	0.5137	0.9089	0.045*	
C11	0.3430 (4)	0.3696 (3)	0.7891 (3)	0.0409 (7)	
H11	0.3522	0.3195	0.8471	0.049*	
C12	0.1896 (4)	0.3268 (3)	0.6649 (3)	0.0398 (7)	
C13	0.1775 (4)	0.4022 (3)	0.5803 (3)	0.0426 (7)	
H13	0.0753	0.3747	0.4973	0.051*	
C14	0.3180 (4)	0.5193 (3)	0.6192 (3)	0.0354 (6)	
C15	0.4389 (4)	0.6917 (3)	0.5467 (3)	0.0340 (6)	
C16	0.4123 (4)	0.7408 (3)	0.4402 (3)	0.0384 (7)	
H16	0.3018	0.7034	0.3621	0.046*	
C17	0.5519 (4)	0.8459 (3)	0.4512 (3)	0.0372 (7)	
C18	0.7171 (4)	0.9011 (3)	0.5690 (3)	0.0415 (7)	
H18	0.8112	0.9723	0.5770	0.050*	
C19	0.7401 (4)	0.8499 (3)	0.6729 (3)	0.0399 (7)	
H19	0.8511	0.8870	0.7507	0.048*	
C20	0.6026 (4)	0.7444 (2)	0.6654 (2)	0.0332 (6)	
N1	0.4419 (4)	0.8470 (3)	0.8590 (3)	0.0480 (7)	
N2	0.6013 (3)	0.7991 (2)	0.8911 (2)	0.0360 (6)	
O1	0.7722 (3)	0.9475 (2)	1.1028 (2)	0.0588 (7)	
O2	0.0591 (3)	0.2089 (2)	0.6320 (2)	0.0560 (6)	
H2	−0.0062	0.1822	0.5509	0.084*	
O3	0.2926 (3)	0.5867 (2)	0.52644 (19)	0.0460 (6)	
O4	0.5213 (3)	0.8911 (2)	0.34392 (19)	0.0501 (6)	
H4A	0.6073	0.9578	0.3637	0.075*	
O1W	0.1874 (3)	0.9043 (2)	0.6353 (2)	0.0548 (6)	
H1A	0.379 (6)	0.820 (4)	0.896 (4)	0.072 (14)*	
H1B	0.481 (4)	0.936 (3)	0.889 (2)	0.091 (14)*	
H1WA	0.2675	0.8615	0.6600	0.16 (3)*	
H1WB	0.1586	0.9439	0.7003	0.13 (2)*	

### Atomic displacement parameters (Å^2^)

**Table:**

	*U*^11^	*U*^22^	*U*^33^	*U*^12^	*U*^13^	*U*^23^
C1	0.0487 (17)	0.0328 (14)	0.0279 (14)	0.0084 (13)	0.0136 (13)	0.0054 (12)
C2	0.0392 (16)	0.0332 (14)	0.0284 (14)	0.0057 (12)	0.0089 (12)	0.0061 (11)
C3	0.0485 (18)	0.0438 (17)	0.0339 (16)	0.0090 (15)	0.0035 (14)	0.0043 (13)
C4	0.0378 (17)	0.063 (2)	0.0484 (19)	0.0163 (16)	0.0036 (15)	0.0157 (16)
C5	0.0401 (18)	0.0547 (19)	0.055 (2)	0.0219 (15)	0.0148 (15)	0.0115 (15)
C6	0.0410 (17)	0.0423 (16)	0.0359 (15)	0.0126 (14)	0.0117 (13)	0.0034 (13)
C7	0.0353 (15)	0.0317 (14)	0.0300 (14)	0.0065 (12)	0.0095 (12)	0.0078 (11)
C8	0.0351 (15)	0.0309 (13)	0.0261 (13)	0.0099 (12)	0.0100 (11)	0.0044 (11)
C9	0.0293 (14)	0.0337 (14)	0.0300 (14)	0.0100 (12)	0.0104 (11)	0.0061 (11)
C10	0.0374 (16)	0.0418 (16)	0.0289 (14)	0.0120 (13)	0.0103 (12)	0.0081 (12)
C11	0.0458 (17)	0.0408 (16)	0.0390 (16)	0.0125 (14)	0.0186 (14)	0.0153 (13)
C12	0.0347 (16)	0.0394 (16)	0.0427 (16)	0.0064 (13)	0.0168 (13)	0.0098 (13)
C13	0.0350 (16)	0.0464 (17)	0.0368 (16)	0.0057 (13)	0.0069 (13)	0.0127 (13)
C14	0.0335 (15)	0.0383 (15)	0.0322 (14)	0.0091 (12)	0.0115 (12)	0.0113 (12)
C15	0.0315 (15)	0.0325 (14)	0.0335 (15)	0.0059 (12)	0.0112 (12)	0.0082 (11)
C16	0.0364 (16)	0.0400 (15)	0.0310 (15)	0.0084 (13)	0.0064 (12)	0.0105 (12)
C17	0.0424 (16)	0.0368 (15)	0.0338 (15)	0.0152 (13)	0.0146 (13)	0.0120 (12)
C18	0.0407 (17)	0.0352 (15)	0.0400 (16)	0.0036 (13)	0.0120 (13)	0.0086 (12)
C19	0.0355 (16)	0.0391 (15)	0.0317 (15)	0.0033 (13)	0.0054 (12)	0.0057 (12)
C20	0.0335 (15)	0.0305 (14)	0.0292 (14)	0.0081 (12)	0.0089 (12)	0.0051 (11)
N1	0.0478 (17)	0.0555 (19)	0.0450 (15)	0.0277 (14)	0.0185 (13)	0.0150 (13)
N2	0.0363 (13)	0.0395 (13)	0.0299 (12)	0.0183 (11)	0.0110 (10)	0.0057 (10)
O1	0.0704 (15)	0.0539 (13)	0.0362 (12)	0.0245 (12)	0.0133 (10)	−0.0055 (10)
O2	0.0523 (14)	0.0523 (13)	0.0484 (13)	−0.0072 (11)	0.0124 (11)	0.0178 (11)
O3	0.0350 (11)	0.0515 (12)	0.0369 (11)	−0.0028 (9)	0.0006 (9)	0.0206 (9)
O4	0.0568 (14)	0.0488 (13)	0.0396 (12)	0.0090 (10)	0.0116 (10)	0.0214 (10)
O1W	0.0552 (14)	0.0654 (14)	0.0384 (12)	0.0146 (12)	0.0160 (11)	0.0133 (11)

### Geometric parameters (Å, °)

**Table:**

C1—O1	1.229 (3)	C12—O2	1.362 (3)
C1—N2	1.356 (3)	C12—C13	1.375 (4)
C1—C2	1.477 (4)	C13—C14	1.384 (4)
C2—C3	1.381 (4)	C13—H13	0.9300
C2—C7	1.388 (4)	C14—O3	1.380 (3)
C3—C4	1.385 (4)	C15—O3	1.377 (3)
C3—H3	0.9300	C15—C16	1.382 (4)
C4—C5	1.391 (4)	C15—C20	1.394 (4)
C4—H4	0.9300	C16—C17	1.381 (4)
C5—C6	1.393 (4)	C16—H16	0.9300
C5—H5	0.9300	C17—O4	1.362 (3)
C6—C7	1.371 (4)	C17—C18	1.396 (4)
C6—H6	0.9300	C18—C19	1.373 (4)
C7—C8	1.523 (4)	C18—H18	0.9300
C8—N2	1.497 (3)	C19—C20	1.389 (4)
C8—C20	1.511 (4)	C19—H19	0.9300
C8—C9	1.513 (4)	N1—N2	1.396 (3)
C9—C14	1.384 (4)	N1—H1A	0.81 (4)
C9—C10	1.400 (4)	N1—H1B	0.89 (3)
C10—C11	1.373 (4)	O2—H2	0.8200
C10—H10	0.9300	O4—H4A	0.8200
C11—C12	1.391 (4)	O1W—H1WA	0.8473
C11—H11	0.9300	O1W—H1WB	0.8723
			
O1—C1—N2	124.0 (3)	O2—C12—C11	117.6 (3)
O1—C1—C2	130.1 (3)	C13—C12—C11	119.4 (3)
N2—C1—C2	105.9 (2)	C12—C13—C14	119.8 (3)
C3—C2—C7	121.4 (3)	C12—C13—H13	120.1
C3—C2—C1	129.7 (3)	C14—C13—H13	120.1
C7—C2—C1	108.8 (2)	O3—C14—C9	122.5 (2)
C2—C3—C4	118.0 (3)	O3—C14—C13	115.0 (2)
C2—C3—H3	121.0	C9—C14—C13	122.4 (3)
C4—C3—H3	121.0	O3—C15—C16	115.0 (2)
C3—C4—C5	120.5 (3)	O3—C15—C20	122.6 (2)
C3—C4—H4	119.8	C16—C15—C20	122.3 (3)
C5—C4—H4	119.8	C17—C16—C15	119.2 (2)
C4—C5—C6	121.2 (3)	C17—C16—H16	120.4
C4—C5—H5	119.4	C15—C16—H16	120.4
C6—C5—H5	119.4	O4—C17—C16	117.5 (2)
C7—C6—C5	117.9 (3)	O4—C17—C18	122.6 (3)
C7—C6—H6	121.0	C16—C17—C18	119.9 (3)
C5—C6—H6	121.0	C19—C18—C17	119.7 (3)
C6—C7—C2	121.0 (2)	C19—C18—H18	120.2
C6—C7—C8	128.1 (2)	C17—C18—H18	120.2
C2—C7—C8	110.9 (2)	C18—C19—C20	122.0 (2)
N2—C8—C20	109.8 (2)	C18—C19—H19	119.0
N2—C8—C9	109.7 (2)	C20—C19—H19	119.0
C20—C8—C9	110.8 (2)	C19—C20—C15	117.0 (2)
N2—C8—C7	99.09 (19)	C19—C20—C8	121.8 (2)
C20—C8—C7	113.7 (2)	C15—C20—C8	121.0 (2)
C9—C8—C7	113.1 (2)	N2—N1—H1A	108 (3)
C14—C9—C10	116.4 (2)	N2—N1—H1B	108.0 (14)
C14—C9—C8	121.5 (2)	H1A—N1—H1B	110 (3)
C10—C9—C8	122.1 (2)	C1—N2—N1	124.5 (2)
C11—C10—C9	122.1 (2)	C1—N2—C8	115.0 (2)
C11—C10—H10	118.9	N1—N2—C8	119.5 (2)
C9—C10—H10	118.9	C12—O2—H2	109.5
C10—C11—C12	119.8 (3)	C15—O3—C14	118.9 (2)
C10—C11—H11	120.1	C17—O4—H4A	109.5
C12—C11—H11	120.1	H1WA—O1W—H1WB	112.6
O2—C12—C13	123.0 (3)		
			
O1—C1—C2—C3	4.5 (5)	C8—C9—C14—C13	−177.9 (2)
N2—C1—C2—C3	−176.3 (3)	C12—C13—C14—O3	179.6 (3)
O1—C1—C2—C7	−175.8 (3)	C12—C13—C14—C9	−0.2 (4)
N2—C1—C2—C7	3.3 (3)	O3—C15—C16—C17	−179.8 (2)
C7—C2—C3—C4	0.6 (5)	C20—C15—C16—C17	−0.2 (4)
C1—C2—C3—C4	−179.8 (3)	C15—C16—C17—O4	179.6 (2)
C2—C3—C4—C5	−0.2 (5)	C15—C16—C17—C18	−0.1 (4)
C3—C4—C5—C6	0.1 (5)	O4—C17—C18—C19	−179.3 (3)
C4—C5—C6—C7	−0.6 (5)	C16—C17—C18—C19	0.4 (4)
C5—C6—C7—C2	1.0 (4)	C17—C18—C19—C20	−0.5 (4)
C5—C6—C7—C8	−178.8 (3)	C18—C19—C20—C15	0.2 (4)
C3—C2—C7—C6	−1.1 (4)	C18—C19—C20—C8	−174.9 (3)
C1—C2—C7—C6	179.2 (3)	O3—C15—C20—C19	179.7 (2)
C3—C2—C7—C8	178.8 (3)	C16—C15—C20—C19	0.1 (4)
C1—C2—C7—C8	−0.9 (3)	O3—C15—C20—C8	−5.2 (4)
C6—C7—C8—N2	178.2 (3)	C16—C15—C20—C8	175.3 (2)
C2—C7—C8—N2	−1.7 (3)	N2—C8—C20—C19	69.3 (3)
C6—C7—C8—C20	−65.4 (4)	C9—C8—C20—C19	−169.3 (2)
C2—C7—C8—C20	114.7 (3)	C7—C8—C20—C19	−40.6 (3)
C6—C7—C8—C9	62.1 (4)	N2—C8—C20—C15	−105.5 (3)
C2—C7—C8—C9	−117.8 (2)	C9—C8—C20—C15	15.8 (3)
N2—C8—C9—C14	106.9 (3)	C7—C8—C20—C15	144.5 (2)
C20—C8—C9—C14	−14.5 (3)	O1—C1—N2—N1	5.6 (5)
C7—C8—C9—C14	−143.5 (3)	C2—C1—N2—N1	−173.6 (3)
N2—C8—C9—C10	−70.9 (3)	O1—C1—N2—C8	174.5 (3)
C20—C8—C9—C10	167.7 (2)	C2—C1—N2—C8	−4.7 (3)
C7—C8—C9—C10	38.7 (3)	C20—C8—N2—C1	−115.3 (3)
C14—C9—C10—C11	0.0 (4)	C9—C8—N2—C1	122.7 (2)
C8—C9—C10—C11	178.0 (2)	C7—C8—N2—C1	4.0 (3)
C9—C10—C11—C12	0.1 (4)	C20—C8—N2—N1	54.2 (3)
C10—C11—C12—O2	178.5 (3)	C9—C8—N2—N1	−67.8 (3)
C10—C11—C12—C13	−0.2 (4)	C7—C8—N2—N1	173.5 (3)
O2—C12—C13—C14	−178.3 (3)	C16—C15—O3—C14	170.9 (2)
C11—C12—C13—C14	0.3 (4)	C20—C15—O3—C14	−8.7 (4)
C10—C9—C14—O3	−179.7 (2)	C9—C14—O3—C15	10.1 (4)
C8—C9—C14—O3	2.3 (4)	C13—C14—O3—C15	−169.6 (2)
C10—C9—C14—C13	0.0 (4)		

### Hydrogen-bond geometry (Å, °)

**Table:**

*D*—H···*A*	*D*—H	H···*A*	*D*···*A*	*D*—H···*A*
O4—H4A···O1W^i^	0.82	1.99	2.790 (3)	165
N1—H1B···O1^ii^	0.89 (3)	2.534 (16)	3.025 (3)	115.4 (19)
O2—H2···O1W^iii^	0.82	1.95	2.760 (3)	170
O1W—H1WA···N1	0.85	2.23	2.883 (3)	134
O1W—H1WB···O1^ii^	0.87	2.06	2.861 (3)	152

Symmetry codes: (i) −*x*+1, −*y*+2, −*z*+1; (ii) −*x*+1, −*y*+2, −*z*+2; (iii) −*x*, −*y*+1, −*z*+1.
